# The Effect of Auriculotherapy on Labor Pain, Length of Active Phase and Episiotomy Rate Among Reproductive Aged Women 

**Published:** 2017-12

**Authors:** Parvin Abedi, Hoda Rastegar, Mahboobeh Valiani, Najimeh Saadati

**Affiliations:** 1 Department of Midwifery, Reproductive Health Promotion Research Center, Ahvaz Jundishapur University of Medical Sciences, Ahvaz, Iran; 2 Department of Midwifery, Nursing and Midwifery Care Research Center, School of Nursing and Midwifery, Isfahan University of Medical Sciences, Isfahan, Iran; 3 Department of Obstetrics & Gynecology, Ahvaz Jundishapur University of Medical Sciences, Ahvaz, Iran; Fellowship Perinatology Ward, Maternal-Fetal Medicine Research Center, Shiraz University of Medical Sciences, Shiraz, Iran

**Keywords:** Auriculotherapy, Labor Pain, Active Phase of Labor, Episiotomy

## Abstract

**Objective:** This study aimed to evaluate the effect of auriculotherapy on labor pain, the length of the active phase, and episiotomy rate among reproductive aged Iranian women.

**Materials and methods:** In this study, 80 women were assigned to two groups: auriculotherapy (n = 40) and control group (n = 40). Auriculotherapy was performed in the earlobe in the Zero, Shen Men, Uterus, Pelvic, Abdomen, Spleen, External genitalia, and Master cerebral regions in the cervix dilation of 4, 6, and 8 cm between uterine contractions. The control group received routine hospital care. The labor pain, duration of the active phase, and rate of episiotomy were assessed in two groups. The independent t- test and chi-square were used for statistical purposes.

**Results:** The mean of labor pain during the active phase was 7.56 ± 0.83 in the auriculotherapy group and 8.43 ± 0.69 in the control group (p < 0.001). The length of active phase was significantly lower in the auriculotherapy than that in the control group (176.2 ± 1 min vs. 342.8±87.2 min, p < 0.001). The rate of normal vaginal delivery (without an episiotomy or perineal tears) was significantly higher in the auriculotherapy group than that in the control group (50% vs. 2.5%, p < 0.001).

**Conclusion:** Auriculotherapy is safe, cost effective and devoid of side effects to reduce the labour pain, length of the active phase and the rate of episiotomy in nulliparous women. This method can be considered as a complementary medicine in labour.

## Introduction

Labor pain is one of the most severe pains and has significant psychological and emotional effects on pregnant women. Women react to labor pain differently (1).

The fear of vaginal delivery due to labor pain is usually the reason for high percentages of women with elective cesarean section. A study in Iran showed that the rate of cesarean is varied from 26% to 60% and in some private hospitals it reaches 87% (2). The majority of reasons for selecting cesarean section was fear of childbirth (39.33%) followed by doctor’s recommendations (28.45) and mother request (12.33%) (3).

In total, one out of five women experience prolonged labor. Prolonged labor is a reason for oxytocin usage and negative labor outcomes such as cesarean section (4). A study of 10,661 nulliparous women showed that those with prolonged labor (first stage >30 h) were more likely to have cesarean delivery (OR: 2.28, 95% CI: 1.92–2.72), higher risk of chorioamnionitis (OR: 1.58, 95% CI: 1.25–1.98), and higher admission of neonate to the neonatal intensive care (OR: 1.53, 95% CI: 1.18–1.97) (5).

There are pharmacological and non-pharmacological methods to reduce pain during labor. However, no single method meets all purposes of pain reduction in all women (6). There is adequate evidence to support some methods for pain reduction during labor such as continuous labor support, baths, intradermal water blocks, and using especial positions by the mother. However, methods such as acupuncture, massage, and hypnosis are required in further studies (7). Breathing techniques and aromatherapy could reduce the duration of the active phase and second stage of labor (8). Acupuncture and acupressure could significantly reduce the labor pain and duration of labor (9-10).

Auriculotherapy is a type of alternative medicine that considers the ear as a microsystem that can reflect disorders in the whole body (11). Some researchers believe that there is a map of the whole body on the ear and the ear can be used for the diagnosis and treatment of diseases in the body (12). In a systematic review that recruited 17 interventional studies for reducing the pain of preoperative, acute and chronic pain, the results showed that auriculotherapy could significantly reduce pain intensity in acute and chronic diseases and analgesic request in perioperative pain (13). The rate of elective cesarean section in some countries is high, and use of non-pharmacological methods can help women tolerate labor pain better. (14) This study was designed to evaluate the impact of auriculotherapy on labour pain, duration of active phase of labor, and rate of episiotomy among nulliparous women.

## Materials and methods

This was a clinical trial on 80 nulliparous women who assigned into two groups of auriculotherapy (n = 40) and control (n = 40). The eligibility defined as low-risk pregnancy, gestational age 37-40 weeks, cephalic presentation, singleton fetus, being in active phase, age 18-35, having, at least one healthy ear, and body mass index 19.8-26.4 kg/m^2^. Women who received analgesia 3 h before the intervention, addict women, women with a history of infertility, and women who used oxytocin for induction of labor were excluded from the study. All eligible women gave written informed consent before data collection. According to the purpose of our study, comparing the mean VAS score in 2 groups, the number of subjects in each group was calculated according to the study of Kindberg et.al (14) ; using α = 0.05, power = 0.9, s_1^2 = 1.2, s_2^2 = 2.7 and d = 1.6.

This study started in November 2014 and completed in May 2015 in two educational hospitals (Sina and Imam Khomeini) in Ahvaz, Iran. A socio-demographic questionnaire and a checklist were used for data collection. This study was conducted in accordance with the ethical standards of the Helsinki Declaration of 1975, as revised in 2000.The protocol of this study approved by the Ethics Committee of Ahvaz Jundishapur University of Medical Sciences (Ref No: ajums.REC.1393.190) and this study registered in the Iranian Randomized Controlled Trial Center (Ref No: IRCT2014101919591N1). All participants gave written informed consent prior to data collection.

The eligible participants were randomly assigned in one of the groups by ratio of 1:1. The allocation of first participant was done by someone else that was not aware of purpose of this study. The statistician, who analyzed the data, was not aware of grouping.

**Figure 1:**
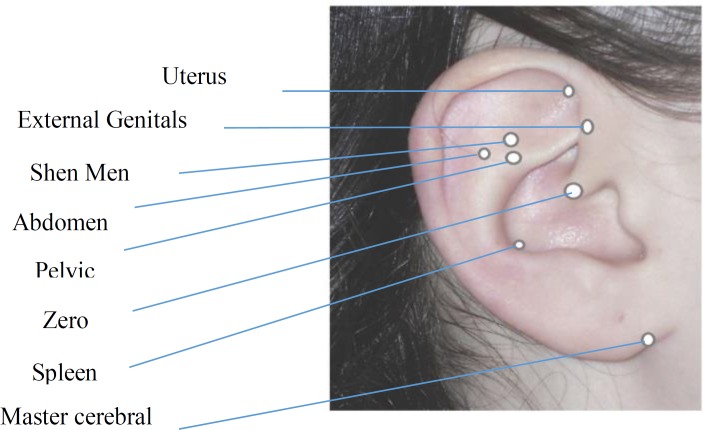
The different points for auriculotherapy (Reproduced from Nogier (1983), with permission)

One of the researchers (HR) trained by a midwife who was expertise in auriculotherapy and had a national certificate (from Iranian Society of Acupuncture and also passed a 6-week training course in France). Then, during the intervention, the researcher took some videos and sent them to the instructor and she checked the accuracy of intervention. Auriculotherapy was performed for women in the intervention group when they had 4 cm dilation by one of the researchers (HR). At first, one ear was disinfected, and in the earlobe in the point of Zero, Shen Men, Uterus, Pelvic, Abdomen, Spleen, External genitals, and Master cerebral auriculotherapy was performed (Figure 1). Each participant in the intervention group received auriculotherapy in the mentioned points when the cervix dilated 4, 6, and 8 cm between uterine contractions. All deliveries were performed by two skilled midwives. 

The control group received standard care according to guidelines of care in Iran. In this way that each two or three women may receive care by one midwife and in the certain time the midwife may perform internal examination or check the fetal heart.

The duration and frequency of uterine contractions were recorded. The length of the active phase and second stage of labor was also recorded. The pain intensity was measured using the visual analog scale and recorded in the checklist for both groups. The score of zero was assigned for those who had no pain and score 7-10 indicated severe pain. The intensity of pain measured in dilations of 4, 6, and 8 cm and the average of them considered as pain intensity. 

The length of the second stage of labor and intensity of pain, mode of delivery, need to episiotomy, newborn’s APGAR in the first and fifth min after birth were recorded for both groups. The control group received the routine care of the hospital. 

All data were entered in SPSS version 21. The Kolmogorov-Smirnov was used to test the normal distribution of continuous data. The independent t-test was used for comparing continuous data while the chi-square test was used for categorical data. The differences were considered significant if p < 0.05.

## Results

**Figure 2:**
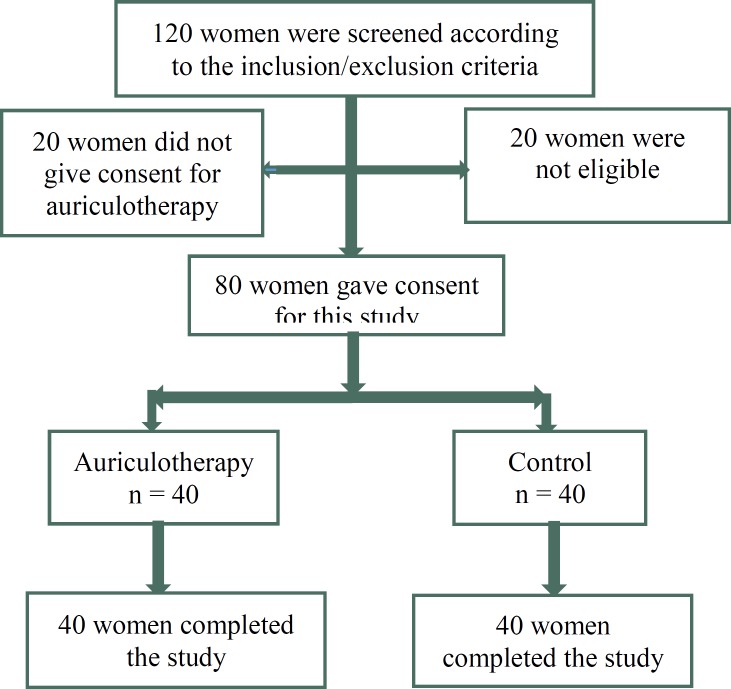
Flow diagram of recruitment and retention of participants in the study

This study included 80 nulliparous women and none of them withdrew during the study (Figure 2). The mean age of women in the auriculotherapy was 24.7 ± 5.1 and in the control group was 27.5 ± 5.1. All women had full term pregnancy. Other socio-demographic characteristics of participants are shown in Table 1.

**Table 1:** Socio-demographic characteristics of women in the two groups of auriculotherapy and control

**Variables **	**Auriculotherapy** **n = 40**	**Control** **n = 40**	**p value**
	**Mean ± SD or N(%)**		
Age (y)	24.7±5.1	27.5±5.1	0.33
Body mass index (Kg/m^2^)	23.7±2.1	22.8±2.2	0.61
Gestational age (wks)	38.6±0.9	38.2±1	0.1
Education			0.96
Illiterate	6(15)	4(10)	
Primary	9(22.5)	9(22.5)	
Secondary	14(34.5)	16(40)	
Diploma	11(27.5)	11(27.5)	
Job			0.5
Housewife	33(82)	34(85)	
Employee	17(17.5)	6(15)	
Economic situation			0.78
Good	11(27.5)	11(27.5)	
Moderate	15(37.5)	18(45)	
Bad	13(32.5)	11(27.5)	

**Table 2:** The labor pain, length of active phase, need to oxytocin and mode of delivery in two study groups

**Variables **	**Auriculotherapy** **n = 40**	**Control** **n = 40**	**p value**
	**Mean ± SD or N(%)**		
Labor painaverage	7.56 ± 0.83	8.43 ± 0.69	< 0.001
Labor pain 4cm	5.35 ± 1.14	6.32 ± 1.09	0.006
Labor pain 6cm	7.15 ± 1.4	7.85 ± 0.92	0.19
Labor pain 8cm	8.35 ± 1.33	9.25 ± 0.86	0.008
Labor pain 10cm	9.42 ± 1.03	9.92 ± 0.26	< 0.001
length of active phase (min)	176.25 ± 101	342.8 ± 87.2	< 0.001
Need to oxytocin (unit)	31.5 ± 4.8	41.25 ± 12	< 0.001
Mode of delivery			
Normal vaginal delivery with intact perineum	20(50)	1(2.5)	< 0.001
Vaginal delivery + episiotomy	7(17.5)	17(42.5)	
Vaginal delivery + 1^st^degree tear	9(22.5)	12(30)	
Normal delivery + 2^nd^degree tear	1(2.5)	6(15)	
Vaginal delivery + 3^rd^degree tear	0	2(5)	
Cesarean section	3(7.5)	2(5)	
Neonate’s weight (gr)	3287 ± 396	3065 ± 444	0.02
Neonate’s sex			
Male	20(50)	26(65)	0.12
Female	20(50)	14(35)	
Apgar at 1minute after birth			< 0.001
4-6	1(2.5)	18(42.5)	
7-10	39(97.5)	22(55)	
Apgar at 5 minutes after birth			0.006
4-6	0	7(17.5)	
7-10	40(100)	33(82.5)	

The mean of pain intensity during the active phase of labor was significantly lower in the auriculotherapy group compared to the control group (7.56 ± 0.83 vs.8.43 ± 0.69, p < 0.001). The length of active phase was significantly lower in the auriculotherapy than that in the control group (176.2 ± 1 min vs. 342.8 ± 87.2 min, p < 0.001). The rate of normal vaginal delivery (without an episiotomy or perineal tears) was significantly higher in the auriculotherapy than that in the control group (50% vs. 2.5%, p < 0.001) (Table 2).

Almost all newborns in the auriculotherapy had APGAR 7-10 at 1 min after birth as compared to the control group (approximately 100% vs. 55%, p < 0.001). All neonates in auriculotherapy had Apgar 7-10 within 5 min of birth as compared to the 82.5% in the control group (p = 0.006) (Table 2).

Only 3(7.5%) of women in the auriculotherapy group and 10(25%) of women in the control group had abnormal postpartum bleeding (p < 0.001). In the auriculotherapy, one baby had respiratory distress while 13(32.5%) of the control group had this problem. There was not any admission in the NICU for newborns in the auriculotherapy group while 8(20%) of neonates in the control group admitted in the NICU (p < 0.001). There were not any side-effects from auriculotherapy in the intervention group.

## Discussion

This study designed to evaluate the effect of auriculotherapy on labor pain, the length of active phase, and rate of episiotomy in nulliparous women. Our results showed that the pain intensity of women in the active stage of labor in the auriculotherapy group significantly reduced compared to the control group. A systematic review including 17 studies on perioperative pain management showed that auriculotherapy was superior to the control in means of acute pain (SMD, 1.56, 95% CI: 0.85-2.26) and use of analgesia (13). A randomized controlled trial was done by Alimi et al., on 90 patients with cancer to evaluate the effect of auriculotherapy on pain intensity. The results showed that auriculotherapy could reduce pain intensity by 36% compared to the patients who received placebo (p < 0.001) (15). We could not find any study that evaluates the effect of auriculotherapy on pain intensity during labor. However, if we consider the pain intensity of labor equal to acute pain or pain resulting from cancer, our results are similar to the results of Asher et al. and Alimi et al. In our study the length of the active phase was reduced significantly in the auriculotherapy as compared to the control group. We could not find any article to address the effect of auriculotherapy on the duration of the active phase. However, a study conducted by Calik & Komurcu (16), to evaluate the effect of SP6 acupressure on labor pain and active phase duration that was conducted on 100 nulliparous women who randomly assigned in two groups of acupressure or routine hospital care. The results showed that women in the acupressure group significantly had lower pain and duration of the active phase (225 min vs. 320 min, p < 0.001). Our results regarding labor pain and duration of the active phase are similar to Calik et al. 

Results of our study showed that although the rate of cesarean section was not significantly different between two groups, the rate of normal vaginal delivery (without an episiotomy or perineal tears) was significantly higher in the auriculotherapy than that in the control group. A case-control study was conducted by Citkovitz et al. (17), on 45 women who underwent acupuncture and 127 women who received routine care of the hospital. The rate of cesarean was significantly lower in the case group compared to the control group (7% vs. 20%, p = 0.004).

The mean of Apgar scores at 1 and 5 min after birth was significantly higher in the auriculotherapy compared to the control group. A study by Borup et al. was conducted on 607 women who received acupuncture, transcutaneous electrical nerve stimulation or routine hospital care. Results showed that mean Apgar score at 5 min after birth was significantly higher in newborns in the acupuncture group compared to other two groups (18). These results are in line with our study. 

A study conducted by Allameh et al (19), to compare the effect of acupuncture and pethidine on labor pain. Results showed that mean of Apgar score at 1 min after birth in the acupuncture group was better than pethidine and control groups, while at 5 min the control group had the highest Apgar score (9.97 compared to 9.90 in acupuncture and 9.77 in pethidine group) (p = 0.08). Our results are similar to Allameh et al., in means of Apgar at 1 min after birth, but discrepant regarding Apgar at 5 min after birth. The reason for this discrepancy may be the fact that Allameh et al., recruited women with their first and second pregnancies (almost 50% of their participants were gravida 2), but we only recruited nulliparous women. 

On the best of researcher’s knowledge, this is the first time that we assessed the effect of auriculotherapy on pain intensity, the length of the active phase, and episiotomy rate of women. The personal characteristics of women may interfere with the response to auriculotherapy that was out of researcher’s control. Lack of placebo is one of the limitations of this study.

## Conclusion

Auriculotherapy is a safe and efficient method to reduce labor pain, the length of the active phase and the rate of episiotomy in nulliparous women. This method can be considered as a complementary medicine in labor.
